# College and Hospital Notes

**Published:** 1910-09

**Authors:** 


### COLLEGE AND HOSPITAL NOTES

The Ernest Wende Hospital is the improved official name, we believe, of what was known formerly as the contagious diseases hospital. The latter name is obnoxious to many people hence a change was desirable. The common council authorised the new designation in memory of Dr. Ernest Wende, late commissioner of health, under whom the first hospital for the treatment of contagious diseases was put in operation, which was during an epidemic of scarlet fever. The present temporary structure will be repaired to serve until the proposed new hospital is complete, to which will be transmitted the name Ernest Wende Hospital.

The Medical Department of the University of Buffalo will begin its sixty-fifth annual session Monday, September 26, 1910. The introductory lecture will be delivered Monday evening, and the regular course will begin Tuesday morning. The University buildings on High Street are commodious structures well adapted to the instruction of medical students. The course covers four years of eight months each. The fees for the four years respectively are $185, $180, $140, $140. The dean is Dr. Matthew D. Mann, 37 Allen Street, and the secretary is Dr. Eli H. Long, at the Faculty Office University Building, 24 High Street.

